# Durability and strength degradation of xanthan gum based biopolymer treated soil subjected to severe weathering cycles

**DOI:** 10.1038/s41598-022-23823-4

**Published:** 2022-11-14

**Authors:** Minhyeong Lee, Yeong-Man Kwon, Dong-Yeup Park, Ilhan Chang, Gye-Chun Cho

**Affiliations:** 1 Disposal Performance Demonstration Research Division, Korea Atomic Energy Research Institute (KAERI), Daejeon, 34057 Republic of Korea; 2 Department of Civil and Environmental Engineering, Korea Advanced Institute of Science and Technology (KAIST), Daejeon, 34141 Republic of Korea; 3 Department of Civil Systems Engineering, Ajou University, Suwon, 16499 Republic of Korea

**Keywords:** Civil engineering, Bioinspired materials

## Abstract

Biopolymer-based soil treatments have shown effectiveness in soil improvement, with successful field-scale implementation. In this study, we explored the effect of cyclic wetting–drying (W–D) and freezing–thawing (F–T) on the strength durability of biopolymer-treated soils. The results indicate that cyclic W–D and F–T gradually degrade soil strength owing to water adsorption and local biopolymer dilution. Poorly graded sand was highly vulnerable to these weathering effects; however, this problem was mitigated when the soil contained a fines content of 15–25%. These biopolymer-treated soils effectively resisted numerous cycles of both W–D and F–T, indicating that biopolymer-treated soils are suitable for earthen slope reinforcement.

## Introduction

Recently, geotechnical engineers have attempted to develop biological soil treatment and ground improvement methods to alleviate environmental concerns related to high CO_2_ emitting cement in geotechnical engineering practices^1^. Among the sustainable approaches for soil treatment and ground improvement, biopolymer-based soil treatment (BPST) has shown sufficient enhancement of geotechnical engineering properties (e.g., strength, permeability control, and erosion reduction) of soil and successful field-scale implementation^2–6^. Furthermore, BPST promotes the seed germination, growth, and drought resistance of vegetation due to the high water retention characteristic and organic origin of biopolymers^7,8^.

**Figure 1:**
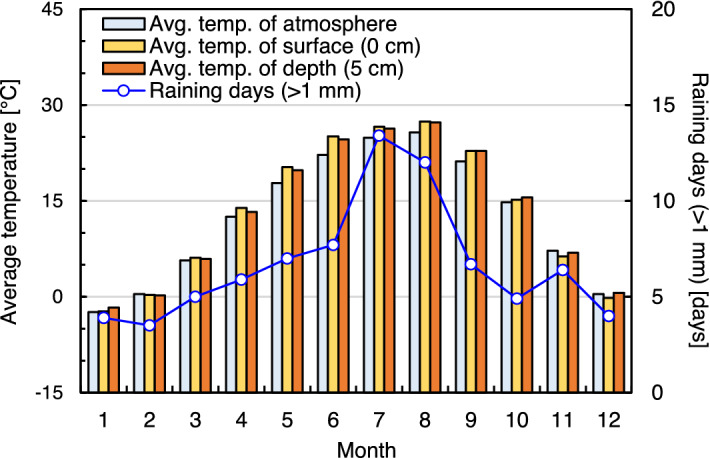
Average air and surface temperatures and frequency of rainfall in South Korea (Seoul) from 1981 to 2010. Data from Korea Meteorological Administration (https://www.weather.go.kr).

Improved geotechnical engineering properties allow BPST to be an environmentally friendly ground improvement method for soil erosion/scouring prevention and slope surface protection^9,10^. However, climate-related weathering processes such as cyclic wetting–drying (W–D) and freezing–thawing (F–T) are suspected to reduce the strength of BPST-reinforced soils^11^. Furthermore, these repetitive weathering conditions can frequently accelerate particle erosion and surface layer detachment, resulting in an unstable vegetative environment. In particular, the heavy rainfall in summer (June to August) and sub-zero temperatures in surface soil during winter (January to February) (Fig. 1) render slope surface reinforcing materials to be inevitably more vulnerable to degradation in South Korea^12^. To assure the reliability of BPST field application, it is necessary to assess the durability of BPST against fluctuating atmospheric weathering conditions.

The ASTM D559 and D560 standard test methods specify the durability assessment methods for soil–cement mixtures exposed to weathering processes, where both standards propose to monitor the mass loss of engineered soil samples after being exposed to 12 cycles of either W–D or F–T^13,14^. However, sample brushing is vulnerable to the diversity of experimental data acquired by different operators^15^. In response, the durability of engineered soil mixtures has been assessed by measuring the unconfined compressive strength (*q*_*u*_) to improve accuracy by reducing experimenter errors^16–21^.

Although some research has evaluated the durability of biopolymer-treated soils by measuring *q*_*u*_ after exposure to cyclic weathering conditions, previous studies mainly focused on W–D weathering^21–25^. However, surface soils usually experience repetitive frosting and thawing, severely impacting the stability and erodibility of soil along slopes during winter seasons in the mid-latitude regions of the northern hemisphere^26,27^. In particular, it has been reported that erosion poses a major risk during the thawing period, where surface runoff can detach particles from the loosened ground surface^28^. Thus, F–T durability analysis should be assessed for BPST. Furthermore, although the soil composition in BPST enhances strength and workability, these responses have not been sufficiently investigated yet.

Therefore, the main objectives of this study were to investigate whether BPST is affected by cyclic W–D and F–T weathering conditions and suggest a more effective BPST composition as a slope reinforcement material. In detail, this study assessed the durability of xanthan gum (XG) and xanthan gum-starch (XS) biopolymer-treated soils, which were applied via wet spraying to embankment slope construction with different fines contents^9^. The durability of XG and XS BPST samples exposed to repetitive W–D and F–T cycles was assessed based on *q*_*u*_, weight loss. The effect of soil composition (i.e., fines content) was analyzed under comprehensive accelerated weathering conditions (i.e., cyclic W–D and F–T) to provide a competitive mixing recipe for field applications.

## Materials and methods

### Materials

#### Soil binders: biopolymers and cement

This study focused on compounds of two gel-type polysaccharide biopolymers; xanthan gum (XG) and starch (ST). Specifically, XG and ST compounds (XS) are a 3:7 mixture of XG and ST, and this formulation has suggested and verified as a slope-soil reinforcing material in the previous studies^8–10,29^, considering their soil strengthening, vegetation promotion, and cost-effectiveness^9^. Combining XG (strengthening purpose) with ST (economic feasibility purpose), this recipe addresses field-applicability enhancement of XG-based soil treatment. In this study, we focused on evaluating the strength durability of XS-treated soil in various soil compositions. In addition, the durability of cement treatment, the most widely commercialized soil improvement method, and pure XG treatment condition were compared with that of XS-treated soils. The detail of individual materials is described below.

XG (CAS: 11138-66-2; Sigma-Aldrich), produced by *Xanthomonas campestris*, is a commonly applied polysaccharide biopolymer in current geotechnical engineering research. XG structure consists of a repeating backbone of 1,4-linked β-d-glucose with side chains composed of two mannose molecules and one glucuronic acid molecule^30^. XG wells form a viscous hydrogel when dissolved in water due to the negative charge of pyruvic and glucuronic acid groups on the side chains contributing to the binding of water molecules^31^. XG is commonly used as a thickener in the food, cosmetics, and petroleum industries and as a soil strengthening agent in geotechnical engineering^32–34^.

ST (CAS No. 9005-25-8, Sigma Aldrich), one of the most commercialized biopolymers, consists of amylose and amylopectin. When dissolved in heated (> 90 °C) water, amylose can induce gelation, forming a firm gel when cooled, whereas amylopectin forms swollen granules that increase the fluid’s viscosity^35^. Several types of ST exist depending on the originated plants (e.g., corn, potato, casaba) and the corresponding ratios of amylose to amylopectin. Because of its cost-effectiveness, combinations of ST and other biopolymers are being investigated for use as soil binding materials^36^. For example, this study used corn ST, which has approximately 25% amylose, to prepare XG and ST compounds (XS) as strengthening materials for levee slope surfaces^9^.

Ordinary Portland cement (manufacturer: Eugene Koryeo Cement Co.) was used in this study for comparison with BPST cases. The cement used had a specific gravity of 3.1, an average grain diameter of 14 μm, and a specific surface area of 2800 cm^2^/g.

#### Sand and Korean residual soil

**Figure 2:**
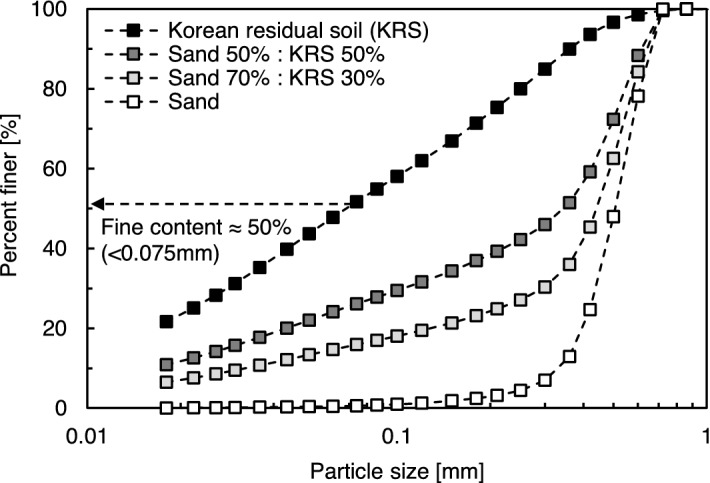
Particle size distribution of sand and Korean residual soil.

Jumunjin sand is a standard sand material in Korea. The sand, which is classified as a poorly graded sand (*SP*) according to Unified Soil Classification System (USCS) criteria, has a mean particle size (*D*_50_) of 0.51 mm and specific gravity (*G*_*s*_) of 2.65. It has a coefficient of uniformity (*C*_*u*_) of 1.12, a coefficient of curvature (*C*_*c*_) of 0.98, and maximum and minimum void ratios (*e*_*max*_ and *e*_*min*_) of 0.89 and 0.64, respectively. The particle size distribution is shown in Fig. 2.

Korean residual soil (KRS), also called *hwangtoh* (in Korean) or red yellow soil, was used to analyze the effect of fines content and simulate in situ soil conditions. KRS is mainly composed of silt with clay and has a *G*_*s*_ of 2.7. KRS is classified as clayey sand (*SC*) based on its particle size distribution (*D*_50_ of 0.07 mm), as shown in Fig. 2. The Atterberg limits of KRS (i.e., liquid limit = 31% and plasticity index = 15) were obtained according to ASTM D4318, as shown in Table 1^37^.

**Table 1:** Details of soil composition and properties used in this study.

Soil composition	Soil [%]		*D*_50_ [mm]	USCS	Liquid limit [%]	Plastic limit [%]	Plasticity index [%]
	Sand	KRS					
Sand	100	0	0.51	SP	–	–	–
Sand-KRS 70:30 mixture	70	30	0.44	SC	21	13	8
Sand-KRS 50:50 mixture	50	50	0.34	SC	27	15	11
Korean Residual soil (KRS)	0	100	0.07	SC	33	18	14

*USCS* Unified soil classification system, *SP* poorly graded sand, *SC* clayey sands.

Before specimen preparation, clean sand and KRS were dried in an oven at 110 °C for 24 h. In this study, jumunjin sand and KRS were mixed with XG- and XS biopolymers. By mixing jumunjin sand and KRS with different mass ratios, four soil compositions were prepared according to riverine embankment design standards (Table 1)^38^. The liquid limit of the prepared sand-KRS mixtures (excluding pure sand) was determined by a fall cone test using a British cone (tip angle 30°, mass 80 g)^39,40^.

### Experimental procedure

#### Preparation of stabilized soil samples

Dry XS compounds were dissolved in deionized water to obtain *m*_*b*_/*m*_*w*_ = 8% (*m*_*b*_/*m*_*w*_ = biopolymer-to-water mass ratio) biopolymer hydrogels. Subsequently, the dried soil was uniformly mixed with the biopolymer solution at *m*_*w*_/*m*_*s*_ (mass ratio of water to soil) = 25%, resulting in biopolymer to soil contents in mass (*m*_*b*_/*m*_*s*_) of 2% with an initial water content of 25%. This initial water content (i.e., water mixing ratio to soil) is the same for all biopolymer-treated soil samples in this study, based on previous research regarding the compaction test of KRS soil and a case study of field application for XS-treated soil^9,41^. In the same way, pure XG-treated soil samples were prepared at *m*_*b*_/*m*_*s*_ of 1% and 2% for sand. In addition, dry cement was dissolved in deionized water at a water-cement ratio of 2.5:1 and then mixed with sand to achieve a cement content of 10% at an initial water content of 25%, representing a typical soil–cement mixture^20^. All binder-soil mixtures prepared in this study are summarized in Table 2.

**Table 2:** Overview of biopolymer-treated soil preparation for durability tests.

Binder types	Content (*m*_*b*_/*m*_*s*_)^a^	Soil composition		Fines content [%]^c^	Label	Average dry density [kg/m^3^]	Average water content [%]^d^
		Sand [%]	KRS [%]				
Xanthan gum and starch^b^	2	0	100	50	XS2(50)	1750	1.7
	2	50	50	25	XS2(25)	1700	1.2
	2	70	30	15	XS2(15)	1640	0.9
	2	100	0	0	XS2	1530	0.6
Xanthan gum	2	100	0	0	X2	1460	0.8
	1	100	0	0	X1	1440	0.6
Cement	10	100	0	0	C10	1610	1.0

^a^*m*_*b*_/*m*_*s*_ = ratio of binder to soil in dry mass.

^b^Xanthan gum-starch composition is 3:7 (0.6% and 1.4%, in total 2% of *m*_*b*_*/m*_*s*_) focusing on slope surface strengthening.

^c^Fines content is estimated based on the particle size distribution of KRS, including 50% of fine particles (< 0.075 mm).

^d^Average water content of specimens after 28 days of dehydration.

The prepared binder-soil mixtures were poured and tamped into a standard cylindrical PVC mold with an inner diameter of 50 mm and a height of 100 mm (i.e., a length-to-diameter aspect ratio of 2). When biopolymer-treated soil is implemented in a field slope as surface protection layer (i.e., 5–10 mm thickness), it is typically exposed to air and dried out via evaporation. To focus on how cyclic weathering environments degrade the dry strength of biopolymer-treated soil after the biopolymer treatment has provided sufficient strengthening effects through air drying; all specimens were equivalently dried at room temperature (23 °C) for 28 days until the soil sample weight was no longer changed^9^. The PVC mold was removed after 7 days to ensure adequate drying in all directions. The average initial dry density and water content after 28 days of drying are listed in Table 2. All samples were prepared in triplicate to obtain reliable average strength values.

#### Cyclic W–D and F–T programs in the laboratory

**Figure 3:**
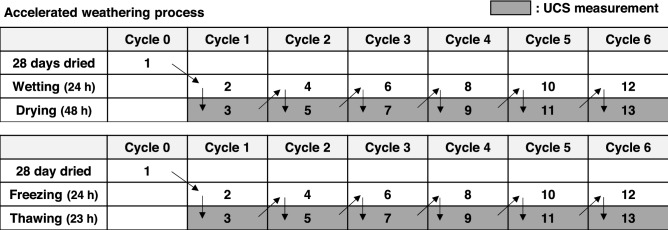
Order of cyclic weathering under laboratory conditions.

**Figure 4:**
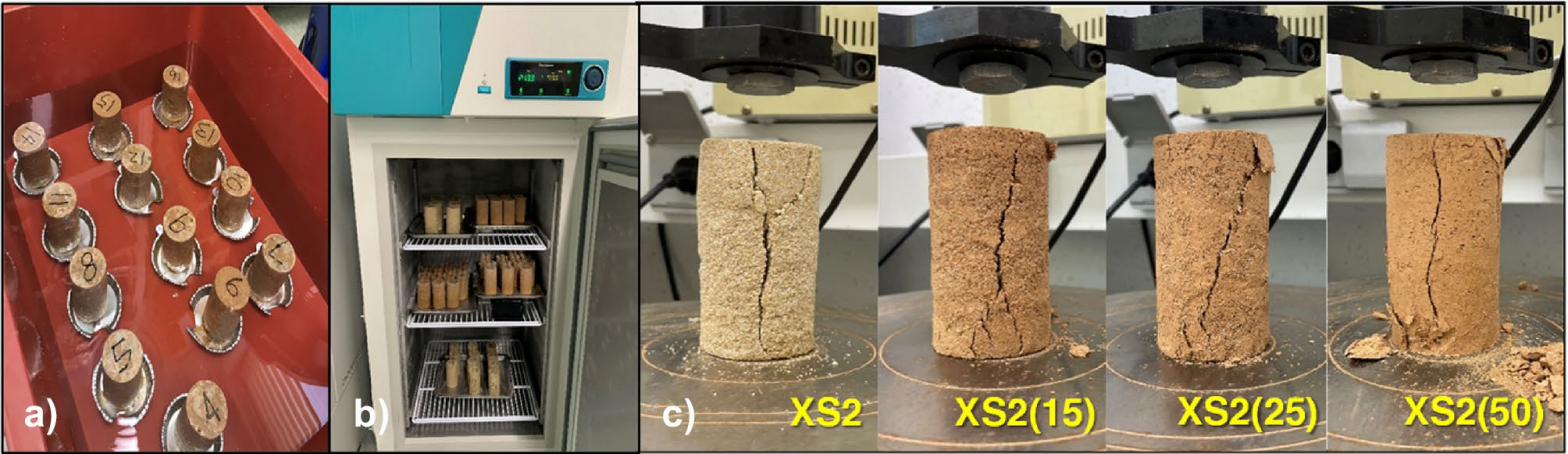
Durability test of biopolymer-treated soil in the laboratory. (**a**) Wetting, (**b**) Freezing (− 20 °C), (**c**) Unconfined compressive strength measurement of XS2, XS2(15), XS2(25), and XS2(50) specimens (controls).

All samples dried for 28 days were exposed to facilitated weathering conditions: cyclic W–D and F–T processes according to the methods suggested in ASTM D559 and ASTM D560. However, the cycle durations specified in the standards were not rigorously followed, as they were considered too short for the biopolymer-treated soil^13,14^ (Fig. 3). ASTM D559 defines one W–D cycle as 5 h of sample immersion followed by a sufficient drying process and suggests at least 12 repeated cycles, equivalent to 60 h of cumulative soaking time^13^. In contrast, this study conducted a W–D cycle of submerging samples in distilled water for 24 h (Fig. 4a), followed by a minimum of 48 h of oven drying at 35 °C until water content reached the previous water content value before immersion, thus simulating a more severe weathering condition. This wetting–drying process was repeated six times, which is equivalent to a total of 144 h of cumulative soaking time.

The F–T cycle consisted of freezing samples at − 23 °C (250.15 K) for 24 h (Fig. 4b) using a laboratory freezing chamber (FMG-300, JEIO Tech.), then thawing for 23 h at 23 °C (room temperature according to ASTM D560^14^. Both the W–D and F–T processes were repeated six times, and the durability of soils was analyzed in terms of *q*_*u*_ and soil weight variation after completing each cycle (Fig. 4c).

#### Unconfined compression test

Unconfined compression tests were performed to assess the *q*_*u*_ and stiffness (*E*_50_) after the number of cycles using a universal loading apparatus (HM-5030.3F, Humboldt). The loading rate of the compression tests was set to 1% strain (1 mm) per minute. Before loading onto the machine, all samples were trimmed to flatten the top and bottom surfaces. The samples were then compressed until failure, and the axial load was obtained automatically every 1 s. The *q*_*u*_ values were obtained using the maximum axial stress represented in the stress–strain curves. The secant modulus of elasticity (*E*_50_) was obtained by measuring the slope between the origin and half of the peak strength (1/2 *q*_*u*_) coordinates for all stress–strain curves.

#### Scanning electron microscope observation

The microstructures of biopolymer-treated soil were observed using scanning electron microscope (SEM) equipment (JSM-IT800, JEOR, Japan). The crushed samples obtained after unconfined compressive test at each cycle were attached to a 50-mm-diameter SEM mount with carbon conductive tabs. The specimens were coated with osmium (O_s_O4) for 10 s under vacuum condition using a plasma coater (OPC-60A).

### Results and analysis

#### Strength of unexposed biopolymer-treated soil after 28 days of drying

**Figure 5:**
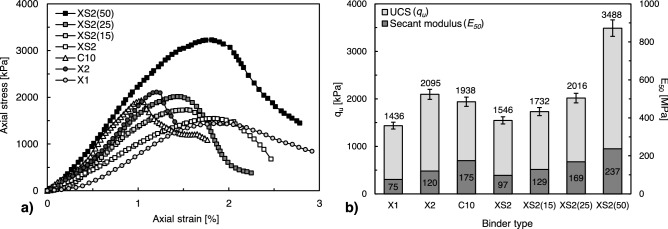
Stress–strain development and strength parameters for controls (0 cycles).

Figure 5A,B show the stress–strain development, unconfined compressive strength (*q*_*u*_), and secant modulus (*E*_50_) of the seven BPST samples dried for 28 days before exposure to the weathering cycle (Table 3). After 28 days of dehydration, the residual water content of all sample cases was less than 2%.

**Table 3:** Strength and stiffness of stabilized soils before and after weathering cycle.

Weathering			Wetting–drying (WD)						Freezing–thawing (FT)					
Samples	Property	0	1	2	3	4	5	6	1	2	3	4	5	6
XS2(50)	*q*_*u*_ [kPa]	3488	2765	1135	650	96	77	68	3382	3295	3379	3233	3158	3133
	*E*_50_ [MPa]	237	125	87	37	7	5	4	210	184	201	217	187	160
XS2(25)	*q*_*u*_ [kPa]	2016	1757	1211	1068	878	732	682	1948	1836	1794	1761	1663	1584
	*E*_50_ [MPa]	169	131	98	80	62	43	24	151	108	95	99	99	97
XS2(15)	*q*_*u*_ [kPa]	1732	1419	1103	810	717	700	639	1637	1587	1520	1457	1321	1299
	*E*_50_ [MPa]	129	98	81	50	50	40	36	119	135	124	121	96	94
XS2	*q*_*u*_ [kPa]	1546	*N/A*^a^	*N/A*	*N/A*	*N/A*	*N/A*	*N/A*	1426	1376	1256	1170	1144	1108
	*E*_50_ [MPa]	97	*N/A*	*N/A*	*N/A*	*N/A*	*N/A*	*N/A*	99	88	82	72	65	59
C10	*q*_*u*_ [kPa]	1938	1686	1479	1302	1307	1200	1060	1782	1660	1540	1538	1485	1400
	*E*_50_ [MPa]	175	136	112	114	104	100	85	178	156	140	138	124	124
X2	*q*_*u*_ [kPa]	2095	*N/A*	*N/A*	*N/A*	*N/A*	*N/A*	*N/A*	2022	1982	1796	1723	1688	1728
	*E*_50_ [MPa]	120	*N/A*	*N/A*	*N/A*	*N/A*	*N/A*	*N/A*	139	126	118	114	110	113
X1	*q*_*u*_ [kPa]	1436	*N/A*	*N/A*	*N/A*	*N/A*	*N/A*	*N/A*	1264	1155	1105	1088	1078	1047
	*E*_50_ [MPa]	75	*N/A*	*N/A*	*N/A*	*N/A*	*N/A*	*N/A*	66	65	57	52	61	52

^a^*N/A* denotes ‘not available’ due to collapse of specimens.

**Figure 6:**
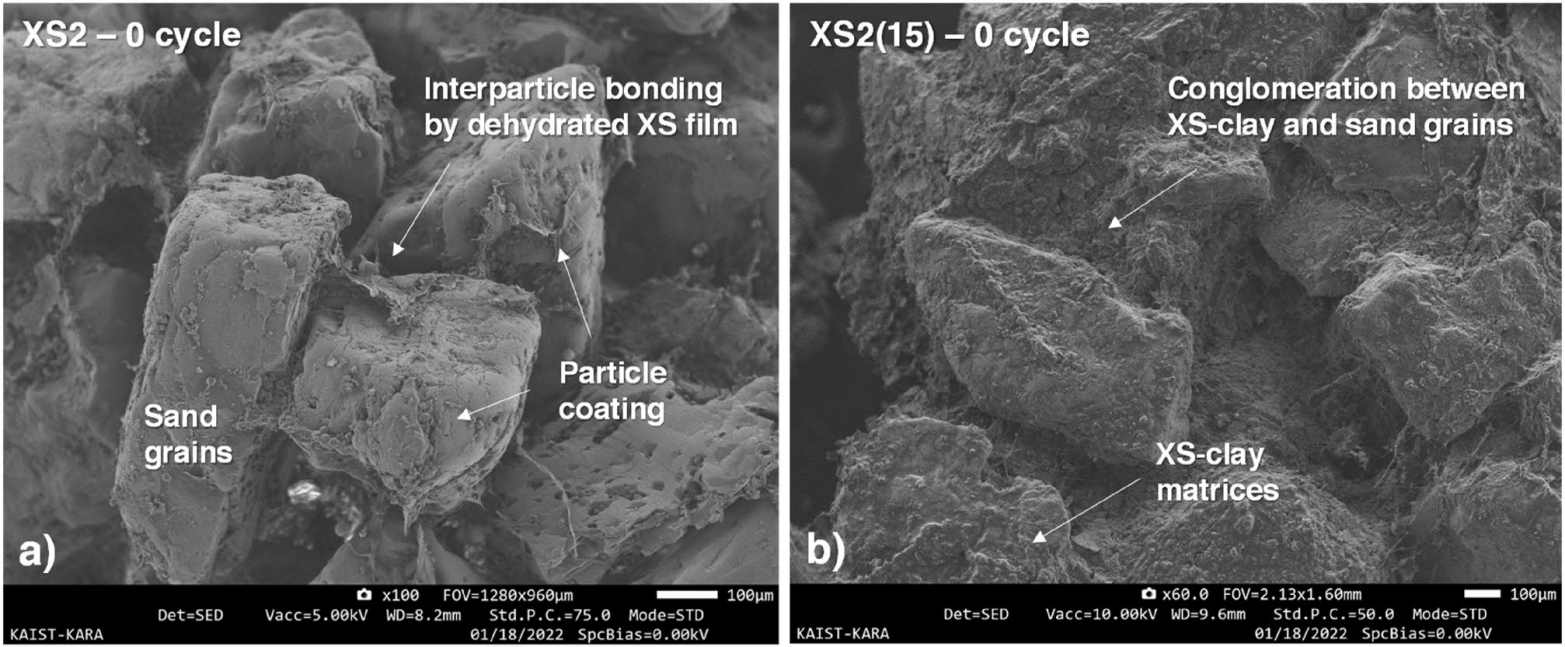
SEM images of unexposed XS-treated soil. (**a**) XS2, and (**b**) XS2(15).

For pure jumunjin sand, X2 (2095 kPa) had the highest initial *q*_*u*_, followed by C10 (1938 kPa), XS2 (1546 kPa), and X1 (1436 kPa) (Fig. 5a). When the XG and XS solution were added to sandy soil, the viscous XG and XS solution contacts the coarse grain surface. They coat the sand particles and form a viscous film that creates bridges between particles^34^. Afterwards, by permitting water evaporation over the curing period, the air dehydration renders XG and XS hydrogels into condensed and stiff films than can provide higher bonding strength^6^ (Fig. 6a). The formation of intergranular matrices amongst sand grains dominantly contributed to the increase in the overall *q*_*u*_ and *E*_50_ of the biopolymer-treated soil. Therefore, the dried biopolymer-treated soil exhibited strength comparable to cement-treated soil, even in cohesionless soil. Furthermore, when dehydrated, a higher XG content was accompanied by a more extensive and thicker XG matrix, causing higher *q*_*u*_ and *E*_50_ values. Even though X2 treatment of sand results in higher *q*_*u*_ than cement treatment, it shows higher ductility with lower stiffness with regard to C10.

The soil composition also affects *q*_*u*_, as it is evident that soil with a higher fines content has higher *q*_*u*_ than pure sand with the same XS content. For example, when 15%, 25%, and 50% fines were included in the soil, *q*_*u*_ increased by 1.1, 1.2, and 2.3 times, and *E*_*50*_ increased by 1.3, 1.7, and 2.4 times for XS2(15), XS2(25), and XS2(50), respectively (Fig. 5b). It is because XS biopolymer can interact directly with clay particles via electrostatic interactions, hydrogen bonding, and ionic bonds owing to the electrical charges on clay surfaces^34^. In other words, biopolymer monomers and fine particles combine to form interparticular bridge resulting a formation of dense and tightly-bonded biopolymer-clay matrices, accompanied by conglomeration effect which biopolymer-clay matrices agglomerating sand grains and behaves like secondary grains between sand particles^3^ (Fig. 6b). Consequently, based on the fact that high dry strength and stiffness are obtained by fine addition at initial state (in an unexposed state), we can anticipate that the fine addition will have a similarly positive effect on the resistance to repeated weathering conditions.

#### Cyclic W–D responses of biopolymer-treated soil

**Figure 7:**
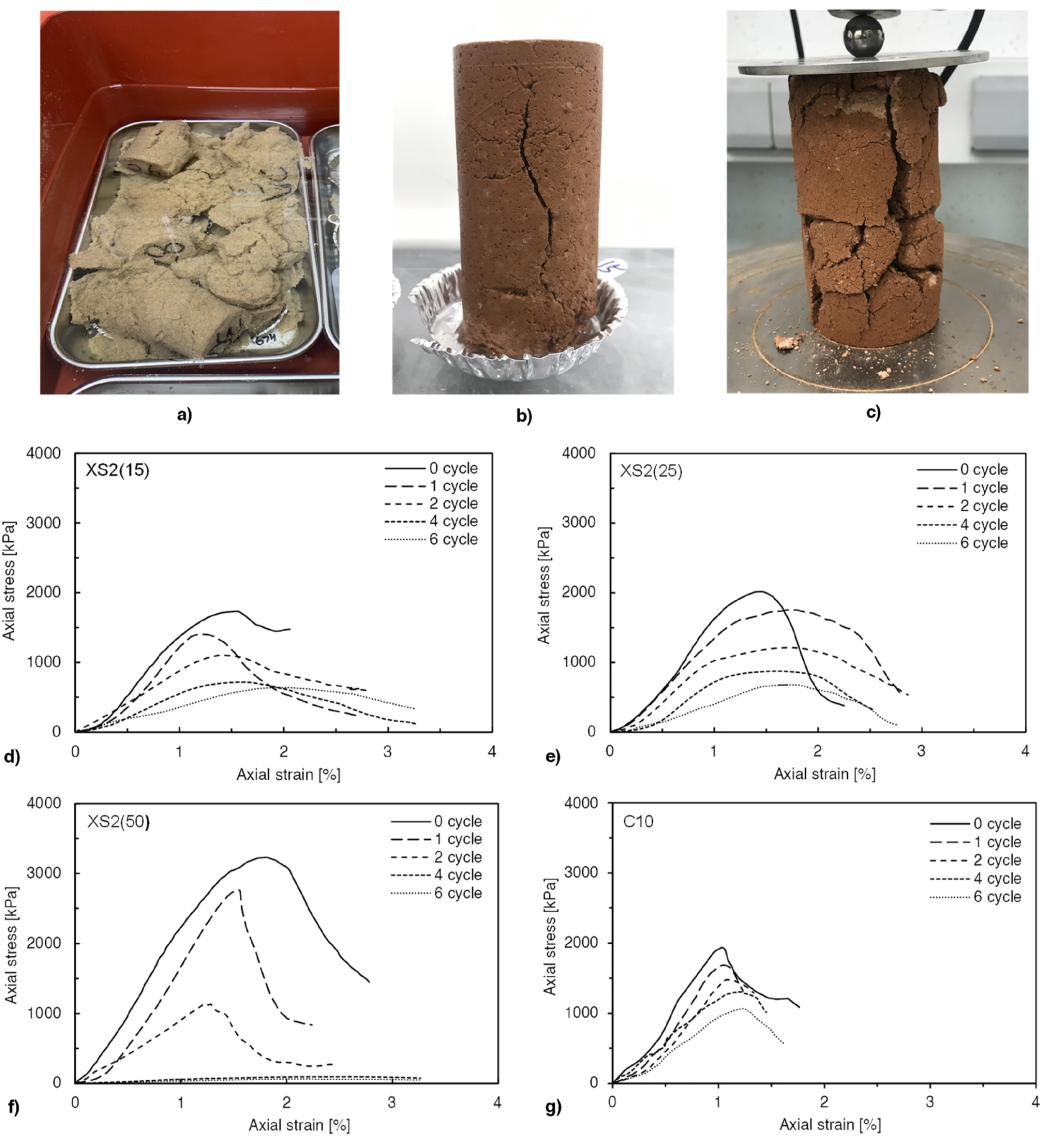
Response to exposure under W–D cycles. (**a**) The collapse of XS2 samples at the first wetting. (**b**) Crack development in XS2(50) after the second W–D cycle. (**c**) XS2(50) splitting into fragments after repetitive swelling and shrinkage (after the sixth cycle). Stress–strain curves after cyclic W–D (1–6 cycles) of (**d**) XS2(15), (**e**) XS2(25), (**f**) XS2(50), and (**g**) C10.

The responses of the BPST samples exposed to cyclic W–D are presented in Fig. 6. Although XG- and XS-treated pure sands (i.e., X1, X2, and XS2) showed competitive strength compared to cement-treated sands (i.e., C10) before exposure to wetting, the X1, X2, and XS2 cases instantly collapsed during the first submerging process owing to their self-weight (Fig. 7a). Thus, the X1, X2, and XS2 conditions were inappropriate for evaluation in the following W–D cycles. When biopolymer-treated pure sand was dehydrated, biopolymers as gel phase filling the pores shrunk and shifted to tensile biofilm, resulting in an interconnected grain structure with voids^34^. Water permeates into the pores when the dried samples are immersed, causing the biopolymer to swell (i.e., a phase shift to a viscous hydrogel state occurs)^42^. The significant swelling caused by the rehydration process, which is mainly induced by the hydrophilicity of the biopolymer, deteriorates the bonding strength, causing samples to collapse under their self-weight.

After repeated W–D cycles, the maximum axial stress values decreased, and the strain corresponding to the maximum axial stress value increased in the other BPST and cement treatment cases (Fig. 7d–g). Specifically, XS2(15) and XS2(25) exhibited higher ductility with increased failure strain (i.e., more than 1% strain) (Fig. 7d,e), which implies that the cyclic disturbances of the biopolymer film increase the possibility of high ductility and strain softening of biopolymer-treated soil^23^. Similar to XS2(15) and XS2(25), XS2(50) showed a rapid reduction in peak stress, whereas the failure strain decreased, implying lower ductility (Fig. 7f). This happened due to the cracks observed after repeated cycles (Fig. 7b,c). In contrast to the biopolymer-treated soils, C10 maintained relatively brittle behavior after cyclic W–D, with failure occurring at approximately 1% strain (Fig. 7g).

**Figure 8:**
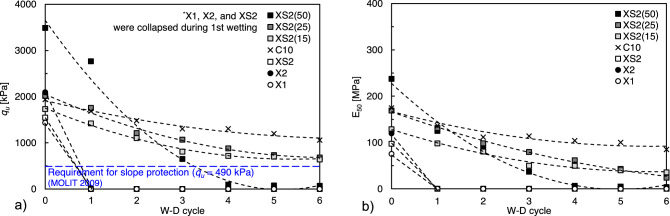
Cyclic W–D durability test result (**a**) *q*_*u*_, (**b**) *E*_50_.

Figure 8 presents the *q*_*u*_ and *E*_*50*_ after the first to sixth W–D cycles. The *q*_*u*_ values of all specimens gradually decreased with the number of W–D cycles (Fig. 8a). In particular, XS2(50), which maintained the highest *q*_*u*_ (2765 kPa) up to the first cycle, exhibited a sharp decrease after the second cycle. Meanwhile, the *q*_*u*_ values of C10, XS2(15), and XS2(25) gradually decreased up to the sixth cycle, whereas these samples maintained a higher *q*_*u*_ than that of XS2(50) after the second cycle. After the sixth W–D cycle, the *q*_*u*_ was the highest in C10, followed by XS2(15), XS2(25), and XS2(50). The *q*_*u*_ values of XS2(15) and XS2(25) satisfied the strength requirement (above 490 kPa) for maintaining vegetation on slope surfaces recommended by the Ministry of Land, Infrastructure, and Transport (MOLIT) of South Korea, even after six cycles^43^. Furthermore, the *E*_*50*_ deterioration of XS2(15) and XS2(25) mainly occurred during the first cycle and became constant after the first cycle (Fig. 8b). In contrast, the *E*_*50*_ value of XS2(50) showed a continuous decrease with W–D cycles, showing a tendency similar to the *q*_*u*_ results.

As previously shown in Fig. 7b,c, a rapid *q*_*u*_ deterioration of XS2(50) was observed due to cracks developed on the surface of the samples despite their high initial strength. During the drying process, cracks are generated on the surface of XS2(50) owing to the extreme shrinkage and swelling caused by moisture loss and absorption. The XG biopolymer has a natural affinity toward the water, originating from the anionic side chains in its molecular structure^30^. Thus, biopolymer-treated soils, especially those that include fines, would attract and retain more moisture within the soil matrix through synergetic effects with clay activity^44^. This characteristic was expected to contribute to more shrinkage, especially in XS2(50), with more fines content. Once a crack occurs on the surface, swelling and shrinkage could be accelerated because water could easily penetrate the samples and react with a larger specific surface than before. With the growth of surface cracks, the XS2(50) samples were eventually split into several fragments, resulting in a *q*_*u*_ less than 1/36 times (after the fourth cycle) the initial strength.

**Figure 9:**
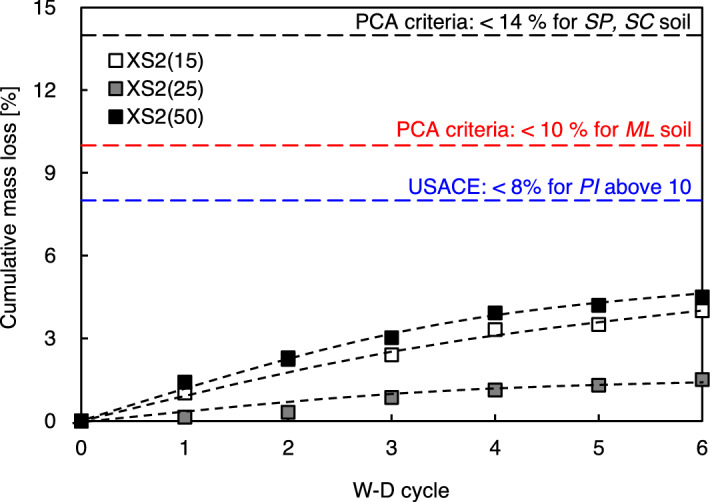
Variation of cumulative mass loss of XS-treated soil by number of W–D cycles.

Figure 9 presents the cumulative soil loss with respect to the initial weight of each sample during the repetition of the W–D process. By the end of the sixth W–D cycle, the XS2(25) samples showed the least weight loss of 1.5%, whereas XS2(15) and XS2(50) showed losses of 4% and 4.5%, respectively. XS2(15) and XS2(50) exhibited detachment of coarse particles at the surface during the submerging process, which indicates that the biopolymer film diluted by repeated exposure to moisture gradually lost its soil binding capabilities. The soil loss that occurred in XS2(50) originated from the detachment at the periphery of the cracks rather than the overall surface. However, all XS-treated soils demonstrated allowable soil loss based on criteria from the Portland Cement Association (i.e., maximum allowable mass loss of 14% for *SP*, *SC* soils) and the United States Army Corps of Engineers (i.e., loss less than 8% for granular soil with a plasticity index above 10)^45,46^.

**Figure 10:**
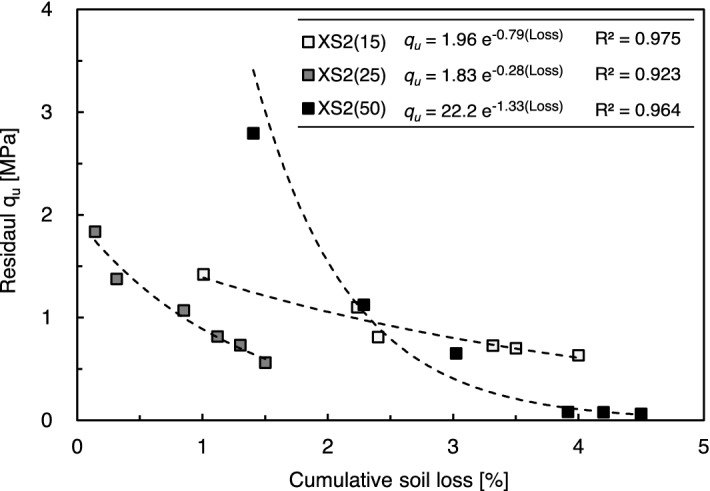
Relationship between cumulative soil loss and residual *q*_*u*_ of biopolymer treated soil.

Figure 10 shows the correlation between the cumulative soil loss amount and *q*_*u*_ of the biopolymer-treated soil after each cycle. The *q*_*u*_—soil loss showed an exponential correlation with high R^2^ values, which aligns with the observations of Baghadadi and Shihata^15^. However, it was demonstrated that biopolymer-treated soil, including soils with large fines (e.g., XS2(50)), can present a biased correlation due to the severe damage through crack generation after six cycles. Thus, the conventional W–D durability evaluation methodologies for soil–cement could be appropriate for biopolymer-treated soil, which includes a 15–25% fraction of fines.

#### Cyclic F–T responses of biopolymer-treated soil

**Figure 11:**
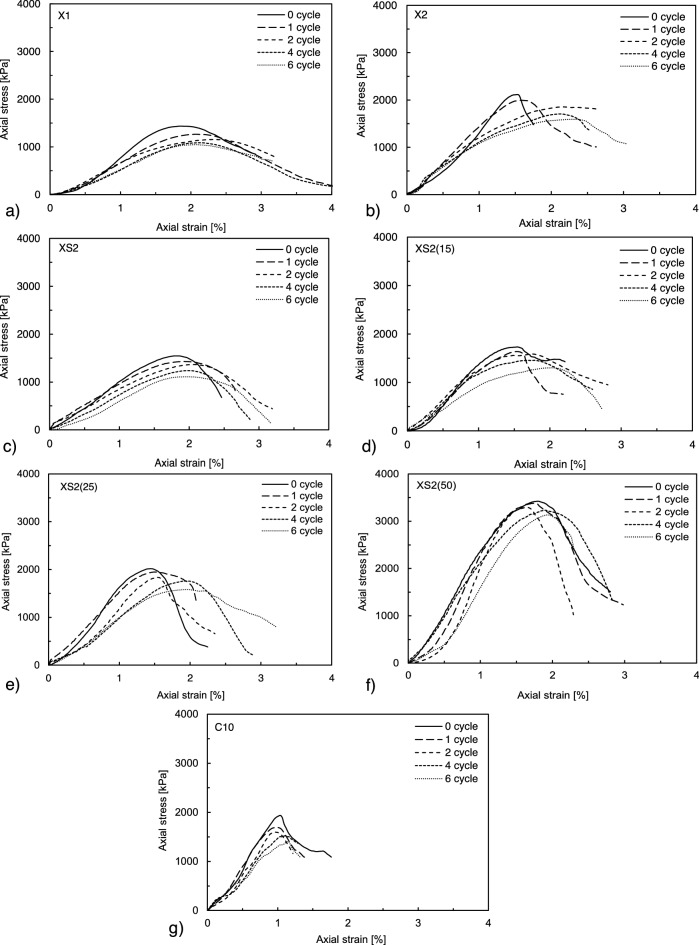
Stress–strain curves after cyclic F–T (1–6 cycles) of (**a**) X1, (**b**) X2, (**c**) XS2, (**d**) XS2(15), (**e**) XS2(25), (**f**) XS2(50), and (**g**) C10.

Figure 11 presents the axial stress–strain curve of all stabilized soils after the repetitive F–T process. As the number of F–T cycles increased, the maximum axial stress values decreased, and the strain corresponding to the maximum axial stress increased (i.e., strain softening) in all stabilized soils (Fig. 11a–g). Although biopolymer-treated pure sands (i.e., X1, X2, and XS2) experienced severe disturbance at the beginning of the W–D process, they exhibited higher resistance against the F–T process without any disturbance compared to the corresponding samples under the W–D weathering condition (Fig. 11a–c). The BPST samples with higher XG content showed more brittle behavior than those with lower XG content at the initial stage, whereas the failure strain increased to over 2% after F–T cycles in both X1 and X2, demonstrating increased ductility as the number of F–T cycles increased. On the other hand, all XS-treated soils presented a strain-softening tendency similar to the others, but the degree of failure strain change between 0 and 6 cycles decreased under higher fines content conditions (Fig. 11c–f). C10 still showed less strain-softening behavior with a smaller failure strain (i.e., approximately 1% of strain) than the biopolymer-treated soils, similar to the W–D result (Fig. 11g). However, the variation in peak stress by the F–T cycle was within the range corresponding to that of biopolymer-treated soils, indicating that the biopolymer-treated soil is competitive with cement-treated soil in terms of F–T resistance performance.

**Figure 12:**
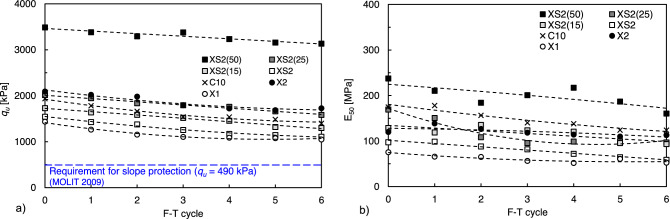
Cyclic W–D durability test result. (**a**) *q*_*u*_ and (**b**) *E*_50_.

The *q*_*u*_ and *E*_*50*_ after each F–T cycle, as shown in Fig. 12, decreased linearly as the number of F–T cycles increased. XS2(50), X2, and XS2(25) maintained higher *q*_*u*_ (3203, 1728, and 1507 kPa) than C10 (1400 kPa) after six cycles, in contrast to the W–D responses. The other biopolymer-treated soils (XS2(15), XS2, and X1) also satisfied the strength requirement (above 490 kPa) recommended for slope stabilization in Korea^43^. Furthermore, the stiffness of the biopolymer-treated soil showed a slight decrease in the same manner as *q*_*u*_. The residual *E*_*50*_ of the biopolymer-treated soil after the F–T process had a range smaller than that of the cement-treated soil, except for XS2(50).

**Figure 13:**
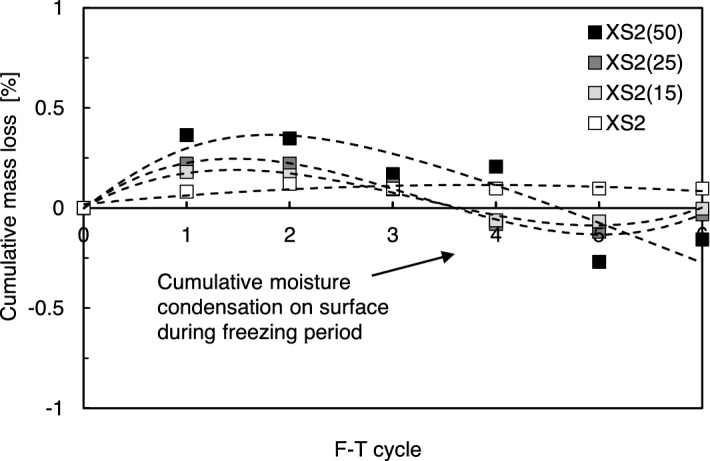
Variation in cumulative mass of XS-treated soil by number of F–T cycles.

The cumulative weight loss trends of the XS-treated soils are shown in Fig. 13. The cumulative soil loss was less than 0.5%, which was less significant than the W–D results, reaching 5% mass loss. A small amount of weight loss occurred in every XS-treated soil after the first and second cycles, but the sample weights slightly increased as the number of F–T cycles increased after the third cycle. This marginal increase in weight could be due to the moisture condensation that occurred on the surface of the samples during freezing. Thus, a correlation between the residual strength and cumulative loss was not observed in the F–T process.

## Discussion

The durability index (*DI*) and strength reduction ratio (*SRR*) (i.e., *SRR* = 1 − *DI*) were considered to analyze the effect of W–D and F–T cycles on the strength characteristics and durability of biopolymer-treated soils. The durability index (*DI*) is the percentage determined by dividing the *q*_*u*_ that remained after the desired number of W–D or F–T cycles by that of an identical specimen subjected to only 28 days of curing^47^;

$$DI \, [\% ] = \frac{{q_{u,N} }}{{q_{u,0} }} \times 100,$$

where *q*_*u,N*_ = UCS after *N* times of cycles; and *q*_*u*,0_ = UCS before exposure to cycles.

**Figure 14:**
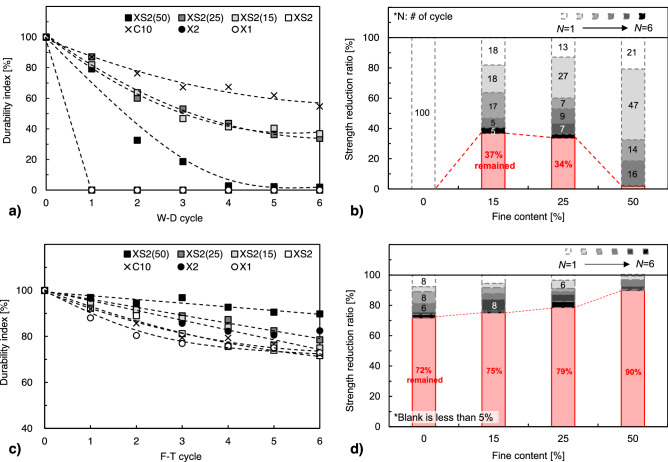
Durability and fines content effect of XS-treated soils under cyclic weathering processes. (**a**) Durability index (W–D), (**b**) strength reduction ratio per cycle (W–D), (**c**) Durability index (F–T), (**b**) strength reduction ratio per cycle (F–T).

Figure 14a,b present the *DI* and *SRR* of XS2 treated soils under W–D, respectively. As mentioned above, the X1, X2, and XS2 samples were excluded because of bulk collapse in the early W–D stages (Fig. 7a). Under W–D weathering conditions, the biopolymer-treated soil generally exhibited unsatisfactory durability performance compared to cement-treated soil. In particular, it showed larger vulnerability in pure sand, and pure KRS which has 50% of clay fraction. Those results is deeply related to the hydrophilic nature of the biopolymer itself, accompanied with high moisture absorption capacity (i.e., higher moisture buffering values) compared to cement^48^.

In pure sand, moisture attraction results in strength deterioration via swelling and local dilution at outermost of biopolymer layer, hindering the viscous biopolymer gel from binding soil particles^23^. Especially, since the electrostatic interaction between sand particles and biopolymer is small, it can be expected that strength degradation is mainly induced by particle detachment at the interface with water (outermost part) when implementing XS biopolymer treatment with poorly graded sand for slope protection.

In pure KRS soil (XS2(50)), although it had the highest initial *q*_*u*_, cracks gradually emerged and showed a significantly low *DI* of approximately 2%. This deterioration behavior is mainly caused by severe swelling and shrinkage induced by synergetic water absorption of both biopolymer and clay activity^33,49^. Therefore, when we implemented slope protection layer using XS biopolymer treatment with fine-rich soil, there is a potential of bulk cracking in layer or the detachment and sliding failure of layer itself to occur at the field.

**Figure 15:**
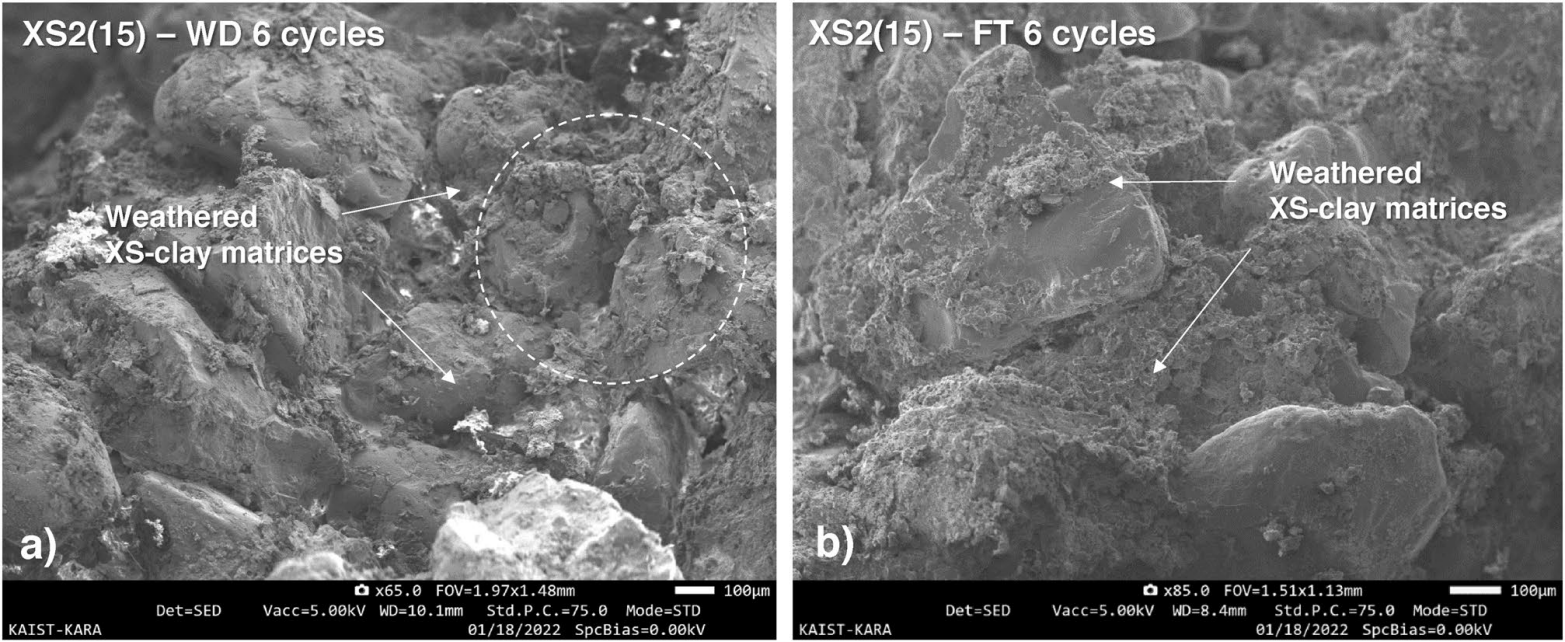
SEM images of XS2(15) after 6 weathering cycles. (**a**) W–D, and (**b**) F–T.

Meanwhile, it confirmed that those vulnerability can be mitigated at soil with 15% and 25% of fine fraction. XS2(15) and XS2(25) showed 37% and 34% *DI* values after six W–D cycles, and the *SRR* decreased from 100% (in pure sand) to less than 18% and 13%, respectively, after the first cycle (Fig. 14a,b). Although repeated W–D process induces outward weathering on XS-clay matrices partially, it can be seen that conglomeration with sand grains is maintained after W–D cycles, showing a durability index of about 35% (Fig. 15a).

In the F–T process, the *DI* of all biopolymer-treated soils showed a gradual decrease, maintaining a range of 72–90% after six cycles (Fig. 14c). Compared to the cement-treated soil retaining 80% DI, the biopolymer-treated soils exhibited competitive durability under F–T weathering conditions. In particular, the addition of fines content remarkably improved F–T durability by reducing the *SRR* from 28% (in pure sand) to 10% (in XS2(50)) after six F–T cycles (Fig. 14d).

Typically F–T durability of stabilized soil (engineered soil) is dominantly affected by the moisture state (i.e., free water in pores), which is related to volume expansion due to ice formation^28,50^. When freezing occurs, the remaining water in the stabilized soil increases in volume by approximately 9%^51^. When the frozen fluids in the pore space expand and reach the available free space in the pore, hydraulic pressure is applied to the stabilized soil matrix, accompanied by a gradual increase in pore size and bond breakage between the binding agent and soil particles^50–52^. However, the biopolymer-treated soils evaluated in this study, dehydrated over 28 days, lost most of the free water occupying the pores through evaporation, resulting in residual water content retention in the range of 0.6–1.7%, as shown in Table 2 (i.e., reduced by 93–98% from the initial water content of 25%). Therefore, disturbance of the soil matrix owing to expanding ice formation in the pore space is tenuous in dried samples. Instead, abrasion on the XS biopolymer-clay matrices could occur the gradual degradation of strength and stiffness. The strength reduction and ductility change under F–T could be attributed to the cumulative abrasion and weakening at biopolymer-clay matrices, which associated by condensed water (i.e., fresh moisture) during each freezing period at surface of the sample (Fig. 15b).

From the comprehensive results of W–D and F–T in this study, it can be noted that SC (clayey sand according to the Unified Soil Classification System) soil containing 15–25% fines content is advantageous for the long-term durability of biopolymer-treated soil. In addition, river design standards in Korea classify soil composition for embankment construction into three grades (I, II, and III), considering leakage resistance and crack prevention. The standards stipulate that a grade I or II soil composition should be utilized for embankment construction^38^. The suggested soil composition from this study satisfied the grade II specification (i.e., well-graded soil with a plasticity index in the range of 7–15), as shown in Table 1. Therefore, it is expected that the slope protection method using biopolymer-treated soil will be sufficiently competitive in terms of strength and durability performance when using SC soil.

However, although the strength and soil loss evaluated in this study are general indicators for assessing the durability of stabilized soil, this controlled environment has not fully demonstrated an in situ climatically degrading environments. Thus, further studies on real weathering effects should be conducted to predict the performance and stability of biopolymer-treated soil in the field. In addition, natural environments contain many microorganisms that may affect the biological stability of biopolymer-treated soil. Therefore, biological degradation due to microbial activity requires further study.

## Conclusion

In this study, the comprehensive strength durability of xanthan gum-based biopolymer-treated soils under cyclic W–D and F–T cycles was evaluated via a series of laboratory experiments. Cyclic W–D of biopolymer-treated soils gradually degrades the strength and stiffness of the soils owing to a high degree of swelling resulting from hydrophilicity during W–D conditions. The vulnerability was very high, particularly in poorly graded sand. However, the resistance to strength deterioration was effectively enhanced when the soil had a certain fines content (15–25%). Meanwhile, strength degradation and soil loss caused by cyclic F–T in a sufficiently dehydrated state were less severe than those caused by W–D, demonstrating competitive performance compared to cement-treated soil. Because 15–25% fine-containing soil shows sufficiently positive effects in both W–D and F–T, satisfying the soil properties in the design criteria for the earthen levee, it is suitable to use biopolymer-treated soil as an earthen slope reinforcement material. However, further studies on durability under natural climate weathering conditions should be conducted to predict long-term performance based on these laboratory results.

## Data Availability

All the experimental data that support the findings of this study are available from the corresponding author upon reasonable request through email.
